# Retraction Note: The epidemiology and chemotherapeutic approaches to the control of urinary schistosomiasis in school-age children (SAC): a systematic review

**DOI:** 10.1186/s12879-020-4820-7

**Published:** 2020-01-31

**Authors:** Tolulope Ebenezer Atalabi, Taiwo Oluwakemi Adubi

**Affiliations:** 1 Department of Biological Sciences, Federal University, Dutsin-Ma, Km 65, P.M.B. 5001, Dutsin-Ma, Katsina State Nigeria; 2 Department of Biological Sciences and Biotechnology, College of Pure and Applied Sciences, Cale University, Imota, Lagos State Nigeria

**Retraction Note: BMC Infect Dis (2019) 19: 73**

**https://doi.org/10.1186/s12879-018-3647-y**

The authors have retracted this article [1] because of methodological inaccuracies and incorrect use of the PRISMA/PROSPERO guidelines of systematic reviews and meta-analyses in the article. A re-examination of the data suggests that the authors’ conclusions are based on a small subset of available literature.

All authors agree with this retraction.
